# Evolving Clinical Features of Diabetic Ketoacidosis: The Impact of SGLT2 Inhibitors

**DOI:** 10.3390/ph17111553

**Published:** 2024-11-20

**Authors:** Selin Genc, Bahri Evren, Onur Selcuk Yigit, Ibrahim Sahin, Ramazan Dayanan, Aleksandra Klisic, Ayse Erturk, Filiz Mercantepe

**Affiliations:** 1 Department of Endocrinology and Metabolism, Konya State Hospital, Konya 42250, Türkiye; selin.genc@saglik.gov.tr; 2 Department of Endocrinology and Metabolism, Faculty of Medicine, Inonu University, Malatya 44280, Türkiye; bahri.evren@inonu.edu.tr; 3 Department of Internal Medicine, Ordu State Hospital, Ordu 52200, Türkiye; onurselcuk.yigit@saglik.gov.tr; 4 Department of Endocrinology and Metabolism, Memorial Sisli Hospital, Istanbul 34384, Türkiye; ibrahimsahin@memorial.com.tr; 5 Department of Endocrinology and Metabolism, Batman Training and Research Hospital, Batman 72070, Türkiye; ramazan.dayanan@saglik.gov.tr; 6 Faculty of Medicine, University of Montenegro, 81000 Podgorica, Montenegro; aleksandranklisic@gmail.com; 7 Primary Health Care Center, Center for Laboratory Diagnostics, 81000 Podgorica, Montenegro; 8 Department of Infection Disease, Faculty of Medicine, Recep Tayyip Erdogan University, Rize 53100, Türkiye; 9 Department of Endocrinology and Metabolism, Faculty of Medicine, Recep Tayyip Erdogan University, Rize 53100, Türkiye

**Keywords:** diabetic ketoacidosis (DKA), euglycemic diabetic ketoacidosis (euDKA), genitourinary infection (GUI), latent autoimmune diabetes in adults (LADA), sodium–glucose co-transporter-2 inhibitor (SGLT2-i), type 1 diabetes mellitus (T1DM)

## Abstract

**Background/Objectives**: The antidiabetic effect of SGLT2 inhibitors (SGLT2-is) is based on their ability to increase glucose excretion through urine by inhibiting the kidney-resident SGLT2 protein. Euglycemic diabetic ketoacidosis (EuDKA) is an uncommon but potentially life-threatening adverse effect of these medications, which are notable for their antidiabetic, cardiovascular, and renal protective properties. This study aimed to clarify the impact of SGLT2-is on demographic, clinical, and biochemical characteristics in patients with DKA. **Methods**: A total of 51 individuals with a diagnosis of DKA were included in the trial; 19 of these patients were treated with SGLT2-is, while 32 were not. Patients diagnosed with DKA and treated with SGLT2-is were compared to those not treated with the medication in terms of clinical, biochemical, and laboratory characteristics. **Results**: The age of patients utilizing SGLT2-is was statistically considerably greater than that of non-users (*p* < 0.001). EuDKA was exclusively noted in the SGLT2-is cohort (*p* = 0.005). Urinary tract infections, vulvovaginitis, and genitourinary infections were substantially more prevalent among SGLT2-i users compared with non-users among both women and the overall patient group (*p* = 0.036, *p* = 0.001, *p* = 0.005, *p* = 0.003, respectively). Plasma glucose concentrations were significantly higher in SGLT2-i non-users (*p* = 0.006). Chloride (Cl^−^) concentrations were elevated among SGLT2-i users (*p* = 0.036). **Conclusions**: The study findings indicate that SGLT2 inhibitors may substantially influence age, serum chloride, EuDKA, and the occurrence of genitourinary infections in individuals with DKA.

## 1. Introduction

Sodium–glucose co-transporter-2 inhibitors (SGLT2-is) are a class of recently developed oral antidiabetic medications that lower blood glucose levels by inhibiting the renal reabsorption of glucose in the proximal tubule [1]. The cardioprotective and renoprotective attributes of these medicines have broadened their application owing to their potential to diminish mortality in diabetic patients suffering from heart failure and chronic kidney disease [2,3,4]. Anti-inflammatory, antioxidant, and immunomodulatory effects may also be achieved by using SGLT2 inhibitors [5,6,7]. Despite the multiple beneficial therapeutic effects of SGLT2 inhibitors, fresh concerns have emerged regarding their potential to elevate the risk of euglycemic diabetic ketoacidosis (euDKA), particularly in individuals with type 1 diabetes mellitus (T1DM) and latent autoimmune diabetes (LADA) [8,9].

Diabetic ketoacidosis (DKA) is a severe acute metabolic complication of diabetes characterized by the excessive synthesis of ketone bodies due to insulin insufficiency, posing a life-threatening risk [10]. Hyperglycemia is a significant indicator in classic diabetic ketoacidosis and is frequently identified promptly. Nonetheless, hyperglycemia may be absent in ketoacidosis associated with the administration of SGLT2 inhibitors [9]. This condition is marked by elevated ketone synthesis despite generally normal blood glucose levels, complicating the diagnosis [11]. Prolonged diagnosis of EuDKA elevates the risk of consequences for patients [12].

The mechanism through which SGLT2 inhibitors enhance ketone synthesis remains inadequately elucidated. It is believed that by decreasing hyperglycemia, they also diminish insulin secretion, increase glucagon secretion, and ultimately promote lipolysis and enhance ketone generation [8,13]. Conditions like T1DM or LADA are believed to induce ketoacidosis in patients due to their reduction of insulin needs for the same rationale [11,14]. The literature underscores that the utilization of SGLT2 inhibitors elevates the likelihood of euDKA, necessitating caution from practitioners about this matter. SGLT2 inhibitors are linked to consequences, including genitourinary infections (GUIs), particularly urinary tract infections (UTIs) and vulvovaginitis, due to their promotion of glucosuria [15,16]. This circumstance markedly heightens the vulnerability to bacterial and fungal infections, particularly in women. The side effects diminish the drug’s efficacy, which is an antidiabetic notable for its cardioprotective and renoprotective attributes, and adversely impact the patient’s quality of life.

This study aimed to investigate the impact of SGLT2 inhibitor administration on clinical, biochemical, and electrolyte parameters in patients presenting to the emergency department with DKA. The potential effects of SGLT2 inhibitor usage on various parameters were assessed by comparing demographic characteristics, laboratory results, and serum electrolyte levels between the groups.

## 2. Results

The general characteristics of the study participants are shown in Table 1. Of the 51 DKA patients, 5 had EuDKA. Table 2 presents the comparative demographic and clinical data of patient groups utilizing and not utilizing SGLT2 inhibitors (SGLT2-is). The age of patients utilizing SGLT2 inhibitors was statistically considerably greater than that of non-users (*p* < 0.001). None of the T1DM patients utilized SGLT2 inhibitors; however, all four patients misdiagnosed as having T2DM at external facilities but subsequently identified as having LADA through comprehensive evaluations at our center were found to be utilizing SGLT2 inhibitors. EuDKA was exclusively noted in the SGLT2-is cohort, and this disparity was statistically significant (*p* = 0.005). Urinary tract infections, vulvovaginitis, and genitourinary system infections were substantially more prevalent among SGLT2 inhibitor users compared with non-users among both women and the overall patient group (*p* = 0.036, *p* = 0.001, *p* = 0.005, *p* = 0.003, respectively).

Table 3 presents the comparative biochemical and hematological laboratory data of patient groups utilizing and not utilizing SGLT2 inhibitors. Plasma glucose concentrations were significantly higher in SGLT2-i non-users (*p* = 0.006). Chloride (Cl^−^) concentrations were elevated among SGLT2-i users (*p* = 0.036). Urine density was similar in both groups; urinary white blood cell count (WBC) was significantly lower in the SGLT2-is group (*p* < 0.001). No notable variation was detected in the other urine, hematological, and biochemical markers. These findings indicate that SGLT2 inhibitors may substantially influence age, serum chloride, and glucose concentrations, as well as the occurrence of urinary infections, in individuals with DKA.

## 3. Discussion

This study investigated the impact of SGLT2 inhibitors on patients with diabetic ketoacidosis, revealing a higher prevalence of euglycemic ketoacidosis in LADA and an increased incidence of genitourinary infections, particularly among female patients. Our findings corroborate analogous studies in the literature and appear to align with prevailing notions indicating that SGLT2-i use may facilitate the onset of EuDKA [17].

The correlation between SGLT2 inhibitors and DKA, as well as EuDKA, is found in the mechanism via which these medications diminish glucose reabsorption in the kidneys and augment glucose excretion in the urine. This may stimulate ketone body formation by enhancing fatty acid use for energy, hence elevating the risk of ketoacidosis [18,19]. It is important to note that glucose levels may be normal or only marginally raised in patients utilizing SGLT2 inhibitors, leading to euglycemic ketoacidosis, which presents a clinical profile distinct from classical diabetic ketoacidosis. The increased occurrence of euglycemic ketoacidosis among patients utilizing SGLT2 inhibitors in our study aligns with this mechanism, a phenomenon commonly highlighted in numerous publications in the literature [9,20,21,22].

The investigation revealed that the incidence of EuDKA escalated with the use of SGLT2 inhibitors in LADA patients. LADA is a form of diabetes characterized by autoimmune properties akin to type 1 diabetes, although it manifests in adults [8]. Endogenous insulin synthesis is diminished, and beta cell functionality is compromised in these patients. SGLT2 inhibitors enhance glucose excretion via urine by inhibiting glucose reabsorption in the kidneys, thereby lowering blood glucose levels through an insulin-independent mechanism. In LADA patients with restricted insulin production, the use of SGLT2 inhibitors may prompt cells to utilize fatty acids as an energy source, resulting in elevated ketone generation [20]. This is the primary mechanism that elevates the risk of developing EuDKA [9]. The elevated occurrence of euglycemic ketoacidosis can be attributed to SGLT2 inhibitors potentially inducing excessive ketone body formation while maintaining normal glucose levels. Symptoms of classical ketoacidosis may be disregarded due to acceptable glucose levels, complicating early identification in LADA patients. The restricted endogenous insulin reserves in LADA patients may render them more susceptible to SGLT2-i administration, hence elevating the likelihood of developing euDKA [8]. An internal medicine specialist rather than an endocrinologist diagnosed all LADA patients in the present study, and they were all under observation at locations other than our own. We believe that because LADA is a kind of diabetes typically observed in adult individuals, it was probably misclassified as T2DM, leading to the use of SGLT2 inhibitors. Considering the literature and this study’s findings, we underscore the necessity of recognizing the potential for LADA and pursuing research in this area, particularly when assessing adult-onset diabetes at the initial diagnosis. Consequently, as demonstrated in the present study, diabetic patients may face lethal consequences from adverse effects arising from the improper use of antidiabetic medications. In conclusion, due to the elevated risk of euglycemic ketoacidosis in LADA patients utilizing SGLT2 inhibitors alongside insulin therapy, these patients require enhanced surveillance to mitigate the risk of ketoacidosis, necessitating more clinical oversight [13].

The higher incidence of genitourinary infections observed in this investigation, as well as in several studies in the literature, may be attributed to the propensity of SGLT2 inhibitors to heighten infection risk, particularly in women, by augmenting glucose excretion via urine [15,16]. Elevated glucose levels in urine foster an environment conducive to bacterial proliferation, hence heightening the risk of genitourinary infections [15]. The prevalence of these conditions, particularly among female patients, can be attributed to the anatomical characteristics of the female genitourinary system, which predispose them to such infections.

An interesting finding in present study is that serum chloride levels were higher in patients using SGLT2-is. SGLT2 inhibitors lower glucose levels by enhancing urinary glucose excretion while also influencing body fluid distribution and electrolyte equilibrium via osmotic diuresis and natriuresis actions [23,24,25]. Chloride is the second most prevalent serum electrolyte following sodium, crucial for regulating body fluids, maintaining electrolyte balance, and ensuring acid–base equilibrium. It is a fundamental component utilized in the assessment of numerous clinical conditions. Studies indicate that chloride is the principal electrolyte governing fluid distribution in the human body based on the likely biochemical nature of solutes. The hypothesis positing that chloride, rather than sodium, serves as the primary electrolyte governing plasma volume in the human body is referred to as the “Chloride theory” [26]. The impact of SGLT2 inhibitors on serum chloride levels remains incompletely elucidated; however, various mechanisms have been proposed, such as the stimulation of the renin–angiotensin–aldosterone system (RAAS), bicarbonate depletion, and proximal tubule effects [26].

**Figure 1:**
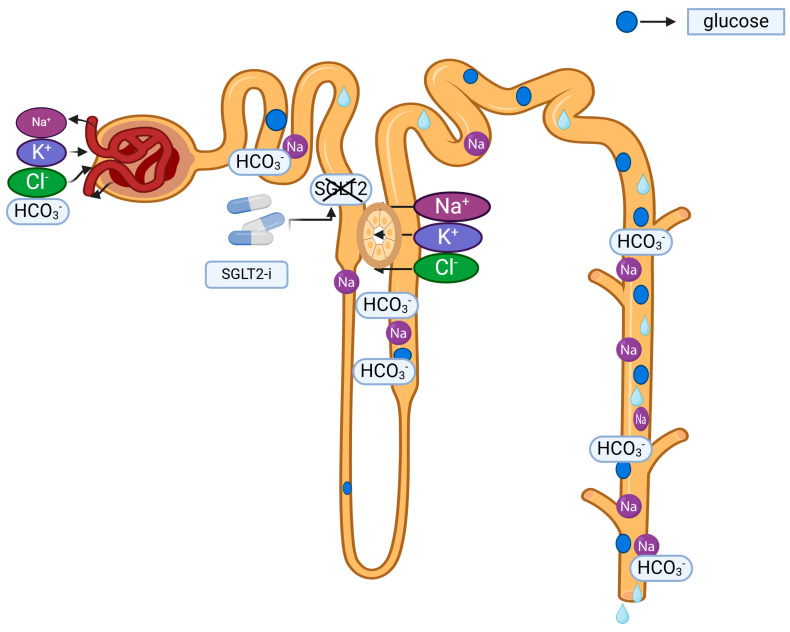
SGLT2-is inhibits SGLT2 in the S1 and S2 segments of the proximal tubule, preventing the reabsorption of sodium, glucose, bicarbonate, and water. SGLT2-is weakens the reabsorption of sodium, glucose, bicarbonate, and water, while enhancing the reabsorption of chloride and potassium. 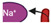: sodium loss, 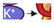: potassium gain, 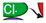: chloride gain, 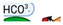: bicarbonate loss, 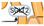: SGLT2 inhibitions by SGLT2-is.

SGLT2 inhibitors are believed to enhance urinary chloride reabsorption through the activation of the RAAS [26]. This may result in the maintenance or elevation of chloride levels. Nonetheless, the impact on the RAAS remains contentious, with several studies indicating that these drugs may suppress RAAS activity [26]. Secondly, SGLT2 inhibitors may diminish serum bicarbonate (HCO_3_^−^) concentrations as a result of buffering organic acids produced during ketone body metabolism. This may result in elevated chloride levels, as the rise in chloride through the bicarbonate–chloride equation could serve as a compensatory mechanism. An increase in blood chloride concentration is anticipated due to the decrease in serum HCO_3_^−^ concentration caused by the buffering of strong organic acid metabolites, such as acetoacetic acid and 3-hydroxybutyric acid, after SGLT2-i therapy [23,24,25]. This also underscores a potential correlation between chloride and ketoacidosis. The study findings may indicate that elevated serum chloride levels in patients utilizing SGLT2 inhibitors corroborate this hypothesis. The limited sample size of the investigation inhibited a comparison of serum chloride levels between euglycemic diabetic ketoacidosis patients utilizing SGLT2 inhibitors and those not utilizing them. It is believed that SGLT2 inhibitors diminish NaHCO_3_ reabsorption by inhibiting the Na^+^/H^+^ exchanger-3 (NHE3) in the renal proximal tubules, while perhaps enhancing chloride reabsorption [24,26,27]. In the investigation, no difference in Na^+^ ion levels was seen between persons utilizing SGLT2-is and those not utilizing them, aligning with existing data [25,28]. The effect of SGLT2 inhibitors on electrolytes and glucose in the nephron is shown in Figure 1. Nevertheless, further research is required to conclusively ascertain which of these pathways is predominantly responsible for this phenomenon or if alternative mechanisms exist.

While SGLT2 inhibitors are typically regarded as safe and advantageous for cardiorenal protection in the management of T2DM, they are linked to EuDKA, an uncommon yet severe consequence [18,29]. This condition is marked by an elevation of ketone bodies (notably β-hydroxybutyrate) and the onset of acidosis, despite the patient’s generally normal blood glucose levels [22]. While elevated ketone generation from fatty acid use due to insulin insufficiency is attributed to the disease, alternative underlying processes may also be present [19,21,30]. In examining the impact of SGLT2-i administration on serum chloride levels in our investigation and the existing literature, we questioned whether this phenomenon could be associated with EuDKA. SGLT2 inhibitors enhance glucose excretion, stimulate fatty acid metabolism, and elevate ketone generation, resulting in decreased serum bicarbonate levels and acidosis [8], which prompted us to consider this condition as the cause of increased chloride levels. Chloride ions may serve as a compensatory response to address the prevailing acidosis through a bicarbonate–chloride balancing mechanism [26]. Alternatively, the converse may also be contemplated. It can be inferred that SGLT2 inhibitors diminish NaHCO3 reabsorption by inhibiting the Na^+^/H^+^ exchanger-3 (NHE3) in the renal proximal tubules while enhancing chloride reabsorption, leading to acidosis due to the reduction in NaHCO3 reabsorption, resulting in ketoacidosis in the absence of hyperglycemia. In EuDKA, the equilibrium of bodily fluids and electrolytes is disturbed [31]. A correlation may exist between the elevation of ketone bodies and chloride reabsorption. The impact of SGLT2 inhibitors on the RAAS system may also lead to electrolyte imbalance and elevate the risk of EuDKA. In conclusion, the capacity of SGLT2 inhibitors to sustain or elevate serum chloride levels may contribute to the acidosis that occurs during EuDKA. Nevertheless, comprehensive investigations examining all these pathways and additional factors are essential for a more definitive understanding.

The current study possesses several limitations, and its deficiencies must be acknowledged during evaluation. The limited sample size, retrospective methodology, insufficient assessment of additional risk factors potentially influencing the onset of EuDKA, variability in treatment protocols, and absence of long-term follow-up are significant factors impacting the results.

This study’s retrospective design makes it difficult to infer cause–effect linkages with certainty. Furthermore, results may not be as robust as those from a prospective design, and the design is susceptible to bias. The results can only be applied to the specific population studied because the sample size was so small (51 patients). Results from studies that include more patients will show how effective SGLT2 inhibitors are. The metabolic differences among this study’s diabetic participants, particularly between type 1 and type 2 diabetes, impact the onset of euDKA and the efficacy of SGLT2 inhibitors. The impact of SGLT2 inhibitors on patients with poor insulin reserves, including those with type 1 diabetes or living with LADA, may vary from that on those with type 2 diabetes. A crucial consideration in assessing the efficacy of SGLT2 inhibitors is, thus, the type of diabetes. Glucosuria, a side effect of SGLT2 inhibitors, raises the danger of genitourinary infections. In addition to altering the clinical course of DKA or its severity, these infections can impact patients’ overall health. This is a significant consideration since it might change the results of this study. Patients on SGLT2 inhibitors had elevated chloride (Cl^−^) levels, according to this research. Although the exact reason for these variations is still unclear, it is possible that other fluid–electrolyte imbalances or acid–base disorders influenced the findings. This condition needs to be clarified by additional study. The results can be skewed due to individual variances in the study’s treatment regimens. How SGLT2 inhibitors work and how the condition progresses in each individual patient may depend on their unique therapy and care regimen. Unfortunately, we do not know much about the SGLT2 inhibitors’ long-term consequences because this study did not follow up with patients for very long. How these medications’ impact on EuDKA or other consequences will evolve over time requires more research.

## 4. Materials and Methods

### 4.1. Study Participants

**Figure 2:**
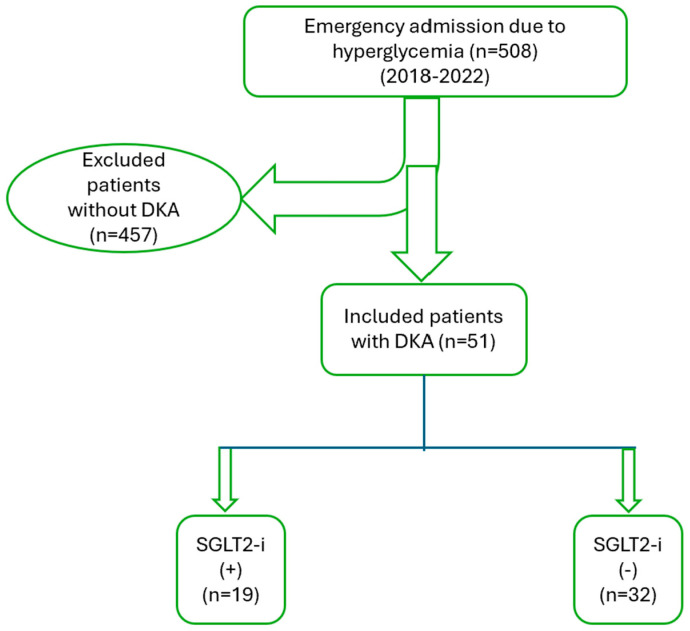
Flow diagram showing the study design. Abbreviations: DKA, diabetic ketoacidosis; SGLT2-is, sodium–glucose co-transporter-2 inhibitors.

This retrospective cohort study was performed in the emergency department of a tertiary hospital from April 2018 to November 2022. The Inonu University Scientific Research and Publication Ethics Committee approved this study (Approval Date: 2 May 2023, Approval Number: 2023/4560). During this research, we meticulously adhered to the guidelines established in the Declaration of Helsinki at every phase. The retrospective nature of this study obviates the need for a voluntary consent form. This study comprised 508 individuals aged 18 and older who presented to the emergency room with hyperglycemia. Among the 508 patients assessed by the endocrinologist in the emergency department for hyperglycemia, 51 were diagnosed with DKA. The patients were classified based on their utilization of SGLT2 inhibitors. This study did not include participants who were under the age of 18, breastfeeding mothers, or pregnant women. The data of all patients were retrospectively gathered from the hospital network system and the national health database. The sociodemographic data, diagnoses, diabetes types, age at admission, medical history, physical examination results, medications administered, and laboratory parameters at admission were processed through this system, anonymized, and documented. Individuals with unspecified diabetic management and missing laboratory results were excluded from this study. A flow diagram of the study design is shown in Figure 2.

### 4.2. Data Collection

When patients were admitted to the emergency department, blood samples were taken to measure plasma glucose, hemoglobin A1c (HbA1c), creatinine, uric acid, aspartate aminotransferase (AST), alanine aminotransferase (ALT), albumin, sodium, potassium, chloride, magnesium, calcium, phosphorus, blood gas, C-reactive protein (CRP), and complete blood count. A full blood count was conducted using a Sysmex XN-1000 automated hematological cell calculator device. A chemiluminescence spectrophotometric method (Beckman Coulter, Brea, CA, USA)) was used to measure the following biochemical parameters: albumin, sodium, potassium, chloride, magnesium, calcium, AST, and ALT, as well as plasma glucose, creatinine, uric acid, and blood urea nitrogen (BUN). HbA1c was measured by the high-performance liquid chromatography method (Adams A1c Ha-8160-BIODPC SN:10912002, Manufacturer: Arkray Factory Inc., 1480 Koji, Konan-Cho, Koka-Shi Shiga, Japan; European Representative Arkray Europe, Prof. J.H. Bavincklaan 51183 At Amstelveen, The Netherlands, made in Japan) [32]. Nephelometric analysis was used to detect CRP (Siemens Healthcare Diagnostics Products GmbH, 35041 Marburg, Germany, Type BN II System, SN: 202826). The emergency department’s ABL 800 flex blood gas instrument was used to perform venous blood gas investigations. Digital imaging of the whole urinalysis flow cell and automated analysis of the urine’s chemistry using dual-wavelength reflectance photometry were employed in the automated urine sediment analysis (BT URICELL 1280–1600 URINALYSIS, Gaziemir, İzmir, Turkey).

A health professional employed a Jadever-Türkter NLD-W 300 kg height and weight scale to collect additional measurements. BMI measurement was calculated as: BMI = weight (kg)/height (m)^2^ [33]. Adjustment for sodium (cNa) was calculated as: cNa = measured Na + (glucose level − 100) × 0.016. Urinary tract infection and vulvovaginitis were confirmed by urine culture and genital examination.

### 4.3. Statistical Analysis

All analyses were conducted using SPSS 22.0 (SPSS Inc. of Chicago, IL, USA). A normality test was conducted before beginning the analyses. Frequency analysis was used to acquire descriptive information regarding this study’s variables. The Student’s t-test and the Mann–Whitney U Test were employed to compare normally and non-normally distributed data between two independent groups, respectively. In 2 × 2 comparisons of categorical variables, the Pearson Chi-square test was employed when the expected value exceeded 5, whereas the Chi-square Yates test was utilized when the expected value ranged from 3 to 5. Fisher’s Exact test was employed when the anticipated value was less than 3. The Pearson Chi-square test was employed for comparisons of categorical variables greater than 2 × 2 when the expected value exceeded 5, whereas the Fisher–Freeman–Halton test was utilized when the expected value was less than 5. The threshold for statistical significance was established at *p* < 0.05.

## 5. Conclusions

The current study indicated that SGLT2 inhibitors may elevate the incidence of euglycemic ketoacidosis in diabetic patients and that genitourinary infections are more prevalent, particularly among women. Moreover, serum chloride concentrations were elevated in patients utilizing SGLT2 inhibitors. These data suggest that caution should be observed for the potential hazards and adverse effects of SGLT2 inhibitors. The prompt identification of euglycemic ketoacidosis and vigilant surveillance of patients predisposed to genitourinary infections is crucial for prevention. Furthermore, this research underscores the need to provide SGLT2 inhibitors to the appropriate patient demographic and accurately differentiate among the many kinds of diabetes. Additional study is required to comprehend the clinical implications of elevated serum chloride levels and their impact on ketoacidosis, as well as cardiovascular and renal consequences.

## Figures and Tables

**Table 1:** General characteristics of the study participants.

Variables	Categorization	*n* (*n* = 51)	Percentage (%)
Gender	Male	18	35.3
	Female	33	64.7
Smoking	Yes	14	27.5
	No	37	72.5
DM Type	T1DM	25	49
	T2DM	22	43.1
	LADA	4	7.8
SGLT2-is	Yes	19	37.3
	No	32	62.7
EuDKA	Yes	5	9.8
	No	46	90.2
UTI	Yes	13	25.5
	No	38	74.5
Vulvovaginitis	Yes	6	11.8
	No	45	88.2
HT	Yes	16	31.4
	No	35	68.6
Hyperlipidemia	Yes	3	5.9
	No	48	94.1
Mortality	Yes	4	7.8
	No	47	92.2
Intubation	Yes	5	9.8
	No	46	90.2
DM Treatment	No Treatment	1	2
	Insulin	28	54.9
	OAD	13	25.5
	Insulin + OAD	7	13.7
	Insulin Pump	2	3.9

Abbreviations: DM, diabetes mellitus; T1DM, type 1 DM; T2DM, type 2 DM; LADA, latent autoimmune diabetes in adults; SGLT2-is, sodium–glucose co-transporter-2 inhibitors; EuDKA, euglycemic diabetic ketoacidosis; UTI, urinary tract infection; HT, hypertension; OAD, oral antidiabetic drug.

**Table 2:** Comparative demographic and clinical parameters of patients according to SGLT2-i use.

Variables		SGLT2-is (+)	SGLT2-is (−)	Total	*p*–Value
Sex	Male	7	11	18	0.859
	Female	12	21	33	
Age (year)		59 ± 18	37 ± 17		<0.001
Height (cm)		164 ± 7	164 ± 9		0.795
Weight (kg)		70 ± 16	67 ± 14		0.525
BMI (kg/m^2^)		26 ± 6	25 ± 5		0.429
Smoking (+)		5 (26.3)	9 (28.1)	14	0.889
DM Type	T1DM	0	25	25	<0.001
	T2DM	7	15	22	<0.001
	LADA	4	0	4	<0.001
EuDKA (+)		5	0	5	0.005 *
HT (+)		6 (31.6)	10 (31.3)	16	>0.05
UTI		8 (42.1)	5 (15.6)	13	0.036 *
Vulvovaginitis		6 (31.6)	0 (0)	6	0.001 *
GUS infection—female		10 (52.6)	5 (15.6)	15	0.005 *
GUS infection—total		12 (63.2)	7 (21.9)	19	0.003 *

* *p* < 0.05 is significant. Abbreviations: SGLT2-is, sodium–glucose co-transporter-2 inhibitors; BMI, body mass index; DM, diabetes mellitus; T1DM, type 1 DM; T2DM, type 2 DM; LADA, latent autoimmune diabetes in adults; EuDKA, euglycemic diabetic ketoacidosis; UTI, urinary tract infection; HT, hypertension; GUS, genitourinary system.

**Table 3:** Comparative laboratory parameters of patients according to SGLT2-i use.

Variables	SGLT2-is (+)	SGLT2-is (−)	*p*–Value
HbA1c (%)	11.2 ± 2.5	10.9 ± 2.2	0.623
Glucose (mg/dL)	398 ± 191	564 ± 206	0.006 *
BUN (mg/dL)	25 ± 13	27 ± 23	0.649
Creatinine (mg/dL)	1.3 ± 0.5	1.6 ± 1.2	0.247
Na^+^ (mmol/L)	135 ± 5	130 ± 6	0.008 *
cNa^+^ (mmol/L)	140 ± 4	137 ± 6	0.205
K^+^ (mmol/L)	4.5 ± 0.8	4.8 ± 0.9	0.176
Cl^−^ (mmol/L)	103 (90−112)	98 (41−113)	0.036 *
Ca^2+^ (mmol/L)	8.9 ± 0.7	8.9 ± 0.8	0.996
Mg^2+^ (mEq/L)	2.3 ± 0.9	2 ± 0.5	0.201
P(mEq/L)	3.9 ± 3.4	4 ± 2.1	0.898
Albumin (mg/dL)	3.6 ± 0.5	3.6 ± 0.9	0.885
WBC (10^3^ µL)	13.5 ± 6.5	14.5 ± 12	0.734
Neutrophil (10^3^ µL)	11.7 ± 6	11.5 ± 10.4	0.935
Lymphocyte (10^3^ µL)	1.4 ± 0.8	2 ± 1.8	0.121
Hemoglobin (gr/dL)	14.2 ± 3.2	13 ± 2.6	0.147
Platelet (10^3^ µL)	261 ± 149	303 ± 115	0.262
CRP (mg/dL)	4.8 (0.3–36)	4.2 (0.3–30)	0.822
pH	7.17 ± 0.17	7.16 ± 0.16	0.853
HCO_3_ (mmol/L)	12.5 ± 5.5	11.7 ± 4.5	0.796
Lactate mmol/L	3.3 ± 3.7	2.6 ± 1.9	0.344
PO_2_ (mmHg)	66 ± 42	69 ± 37	0.733
O_2_ Saturation (%)	97 ± 3	97 ± 2	0.232
Urine Density (g/cm^3^)	1019 ± 7	1019 ± 7	0.831
Urine WBC (/HPF)	16 (1−70)	22 (0−617)	<0.001 *
Urine RBC (/HPF)	12 (0−51)	9 (0−75)	0.178

* p < 0.05 is significant. Abbreviations: SGLT2-is, sodium–glucose co-transporter-2 inhibitors; BUN, blood urea nitrogen; Na, sodium; cNa, corrected sodium; K, potassium; Cl, chloride; Ca, calcium; Mg, magnesium; P, phosphorus; WBC, white blood cell; CRP, C-reactive protein; HCO_3_, bicarbonate; PO_2_, partial pressure of oxygen; RBC, red blood cell.

## Data Availability

All data generated or analyzed during this study are included in this article. The data will be available upon reasonable request (contact person: filiz.mercantepe@saglik.gov.tr).
